# ICP-MS Determination of 23 Elements of Potential Health Concern in Liquids of e-Cigarettes. Method Development, Validation, and Application to 37 Real Samples

**DOI:** 10.3390/molecules26216680

**Published:** 2021-11-04

**Authors:** Andrea Mara, Ilaria Langasco, Sara Deidda, Marco Caredda, Paola Meloni, Mario Deroma, Maria I. Pilo, Nadia Spano, Gavino Sanna

**Affiliations:** 1 Dipartimento di Chimica e Farmacia, Università degli Studi di Sassari, Via Vienna 2, 07100 Sassari, Italy; a.mara@studenti.uniss.it (A.M.); ilangasco@uniss.it (I.L.); saradeidda96@tiscali.it (S.D.); paola94meloni@gmail.com (P.M.); mpilo@uniss.it (M.I.P.); nspano@uniss.it (N.S.); 2 AGRIS Sardegna, Loc. Bonassai, S.S. 291 Km 18.6, 07100 Sassari, Italy; mcaredda@agrisricerca.it; 3 Dipartimento di Agraria, Università degli Studi di Sassari, Viale Italia 39/a, 07100 Sassari, Italy; mderoma@uniss.it

**Keywords:** e-cigarettes, e-liquids, toxic elements, trace elements, ICP-MS

## Abstract

The lack of interest in the determination of toxic elements in liquids for electronic cigarettes (e-liquids) has so far been reflected in the scarce number of accurate and validated analytical methods devoted to this aim. Since the strong matrix effects observed for e-liquids constitute an exciting analytical challenge, the main goal of this study was to develop and validate an ICP-MS method aimed to quantify 23 elements in 37 e-liquids of different flavors. Great attention has been paid to the critical phases of sample pre-treatment, as well as to the optimization of the ICP-MS conditions for each element and of the quantification. All samples exhibited a very low amount of the elements under investigation. Indeed, the sum of their average concentration was of ca. 0.6 mg kg^−1^. Toxic elements were always below a few tens of a μg per kg^−1^ and, very often, their amount was below the relevant quantification limits. Tobacco and tonic flavors showed the highest and the lowest concentration of elements, respectively. The most abundant elements came frequently from propylene glycol and vegetal glycerin, as confirmed by PCA. A proper choice of these substances could further decrease the elemental concentration in e-liquids, which are probably barely involved as potential sources of toxic elements inhaled by vapers.

## 1. Introduction

Electronic cigarettes (e-cigarettes) were introduced on the market in the mid-2000s and are considered a healthier alternative to traditional smoking. There are different types of devices, components, and e-liquids [1,2] to meet all consumer needs. However, all of them share the same components as well as principle of functioning. It consists of a power source, such as a rechargeable lithium battery, and a cartridge (or a tank) containing the liquid (henceforward called e-liquid). An electrical resistance (i.e., the atomizer), activated by a puff or by a button, is the heating element, which promotes the nebulization of the e-liquid and the formation of the aerosol inhaled by the vaper. An electronic system may be able to report the information related to the nebulization process (volts, watts, ohms, puffs) from a smartphone, LEDs, or an integrated screen. The flavored e-liquid is mainly formed by low amounts of water and two EU food additives, namely propylene glycol (E1520, according to the EU classification, (PG)) [3] and vegetal glycerin (E422, (VG)) [4]. Both generally recognized as safe (GRAS) by the U.S. Food and Drug Administration. In addition, it may contain variable amounts of nicotine, never exceeding the concentration of 20 mg cm^−3^ in EU countries [5].

Although they are considered healthier than traditional cigarettes, owing to their lack of toxic products formed by combustion [6,7,8,9], the health risks related to the use of e-cigarettes have also been widely studied [10,11,12,13]. The apparatuses mainly involved are the respiratory [14,15], the cardiovascular [16,17], the nervous [18], and the reproductive systems [19]. Nicotine [20] is likely one of the most toxic components assimilated by means of tobacco smoke. It is quickly assimilated [21] and adversely affects respiratory, cardiovascular, renal, and reproductive systems [22]. However, the presence of nicotine in e-liquids is optional, and it may be dosed to accomplish any nicotine replacement therapy needs. In a close analogy to what happens with traditional tobacco smoke, attention has been paid to the formation of potentially harmful compounds from the thermal degradation of the main ingredients of e-liquids, such as the flavors, PG and VG [23,24,25]. Hence, chromatographic methods devoted to determining species such as nitrosamines, VOCs, carbonyls, polycyclic aromatic hydrocarbons, and aldehydes have been developed for this purpose [26,27,28,29]. Nevertheless, and only with some exceptions [30], the absence of any combustion process and the low temperatures measured in the nebulization process of e-liquids greatly reduce the concentration and the number of harmful species in comparison to what is observed in the smoke of traditional cigarettes [7]. Moreover, the concentration of toxic metals and metalloids contained in the aerosol of e-cigarettes seems lower than that measured in traditional smoke [31,32,33]. Although, Badea et al. measured detectable amounts of rare earths in the serum of e-cigarette smokers [34]. Moreover, in this case, many analytical methods for the determination of elements in aerosols and—more rarely—in e-liquids were recently developed [35]. Only in a few cases, elemental determination is carried out on both liquids and aerosols [25,36,37,38]. In these contributions, the concentration of the elements measured was higher in aerosols rather than in e-liquids, with the only exception being the study performed by Beauval et al. [25], where the amount found in both matrices was comparable. The elemental amounts found in these studies are hardly comparable among them, due to the great differences in e-cigarettes, e-liquids, and sampling techniques and puffing protocols in the aerosol determination [39,40].

Moreover, the analytical technique used for elemental determination is a possible reason for the differences observed among the literature studies. Inductively coupled plasma (ICP) methods (e.g., ICP-mass spectrometry (ICP-MS) [7,25,33,36,37,38,39,40,41,42,43,44,45,46] or ICP-optical emission spectroscopy (ICP-OES) [47,48,49,50]) are the instrumental techniques mainly used for this purpose, although sometimes X-ray fluorescence methods have also been used [51]. As far as the toxic elements are concerned, those most frequently measured are As [7,25,36,37,39,41,42,43,44,45,49,51], Pb [7,25,33,36,37,41,43,44,45,47,51], Cd [7,25,33,36,37,41,42,43,44,45,51], and Cr [7,25,33,36,39,41,43,44,45,47,48,51], while sometimes also Sn [36,39,44,45,47,48,49], Sb [25,36,39,41,49], Hg [25,41,44,45], and Tl [25,41] were quantified.

Although it may seem obvious that determining the amounts of elements that are potentially health-threatening contained in e-liquids might be important in defining the overall level of exposure of vapers, until now the limits posed (or suggested) by countries [52,53] or international organizations [54] on the concentration of toxic elements are quite rare. This also reflects the scarce number of literature contributions addressed to this aim. From a purely analytical viewpoint, it is surprising that the possibility of a severe bias in the ICP-MS determination of these analytes, caused by a matrix almost entirely formed by organic species such as e-liquids, was considered only by one research group [41]. In addition, only two research groups [25,36,41] attempted to quantify a reasonable number among the toxic elements and oligoelements while, to the best of our knowledge, toxic elements such as Ba, Bi, and U were never quantified in e-liquids.

Therefore, the principal aim of this contribution was to develop and validate an ICP-MS method able to determine the total amount of the 23 elements of potential health concern, i.e., Al, As, B, Ba, Be, Bi, Cd, Co, Cr, Cu, Fe, Hg, Li, Mn, Mo, Ni, Pb, Se, Sb, Sn, Tl, U, and Zn, in a reliable sampling of e-liquids produced in Sardinia, Italy. Particular attention has been taken to optimize critical phases such as sample pre-treatment and quantification method. Samples were analyzed simply after a proper dilution or after microwave-assisted mineralization, whereas external calibration (in the absence or in the presence of the matrix) and internal calibration were kept into consideration for quantification. Since the need for studies aimed to evaluate the role of the flavor components in the contamination by trace elements in e-liquids was evidenced in previous publications [25], the optimized methods were tested on 37 different flavors and on the constituents of all e-liquids (i.e., PG, VG, water, and nicotine).

## 2. Results and Discussion

### 2.1. Method Assessment

#### 2.1.1. Sample Pre-Treatment

The technique of sample pre-treatment most frequently described in the literature for e-liquids [25,36,37,41] is a simple dilution in an HNO_3_ aqueous solution, exploiting their complete solubility in such a solvent. However, the e-liquids may represent in principle a strongly interfering matrix in ICP-MS measurement, due to their very high amount (always over 95% *w/w*) of organic matter. Consequently, low levels of dilution of e-liquids also potentially allow for quantifying the trace elements, but inevitably increase the noise and the interference of the carbon-based polyatomic ions (especially on the quantification of elements such as Cr and V [41]). On the other hand, high levels of dilution reduce bias due to matrix effects, but likely permit the quantification only of the most abundant elements. In order to allow a comparison with literature data, the first approach of sample pre-treatment chosen in this study was the dilution of e-liquids, according to Beauval et al. [25,41]. The same method was also used for pre-treating the pure organic constituents of the e-liquids (i.e., PG, VG, and nicotine). Hence, ca. 0.3 g of sample, exactly weighted on an analytical balance (±0.0001 g uncertainty), was diluted to a final volume of 15 cm^3^ with an aqueous solution containing a 2% (*v/v*) HNO_3_ solution and 0.1% (*v/v*) of Triton^TM^ X-100. In addition, by weighting 1.5 g and 0.15 g of the sample (finale volume 15 cm^3^), 1:10 (*w/v*) and 1:100 (*w/v*) dilutions were also tested to optimize the method. All samples were filtered through a 0.22 μm nylon filter prior to ICP-MS analysis. Unfortunately, a high number of ionic counts for blanks were observed for several analytes (i.e., for Cr, V, and Zn) when the 1:10 and 1:50 dilutions were used, likely due to a very strong interference by polyatomic ions. Conversely, using a 1:100 dilution, this interference was reduced, but it was no more possible to quantify the trace analytes. In addition, the high amount of organic species conveyed to plasma causes serious instrumental issues, such as a thick deposit of soot in the RF coil, in the torch, and in the cones. For these reasons, after some preliminary tests, the pre-treatment for dilution was abandoned.

**Figure 1:**
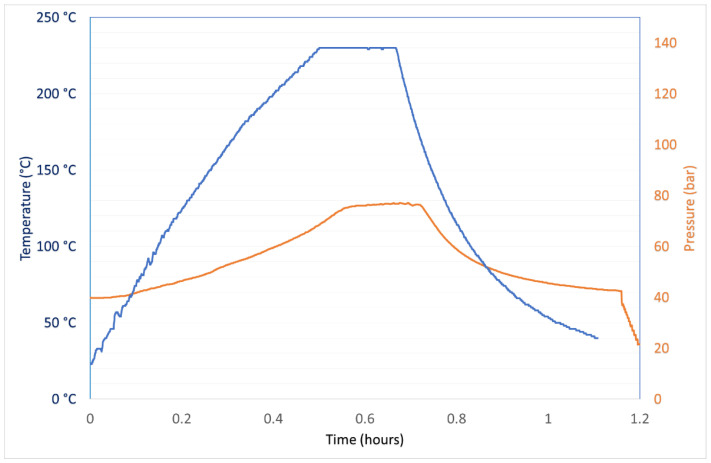
Trends of temperature and pressure inside vessels along a typical mineralization cycle of 0.3 g of an e-liquid dissolved in 0.5 cm^3^ of HNO_3_ and 6 cm^3^ of water.

Another option for the decomposition of the organic matrix in the e-liquids is an acid/oxidant attack assisted by microwaves. An amount of 0.3 g of the sample, exactly weighted on an analytical balance, was treated with 0.5 cm^3^ of HNO_3_ and 6 cm^3^ of water inside a 15 cm^3^ internal volume polytetrafluoroethylene (PTFE) vessel. Due to the high amounts of polyols contained in this matrix, the operative conditions used were the best compromise among the maximization of the oxidizing power and the minimization of the risk of unpredictable and potentially violent reactions inside the vessels. Figure 1 shows the trends of the microwave power and of the internal temperature and pressure of the vessel.

The whole mineralization cycle lasts 70 min. After this time, the vessels were opened at room temperature and the mineralized samples were diluted up to 15 cm^3^ and filtered through a 0.22 μm nylon filter.

#### 2.1.2. ICP-MS Method

Table 1 reports the instrumental parameters and the elemental settings used for the ICP-MS determination of 23 toxic elements and oligoelements in e-liquids.

**Figure 2:**
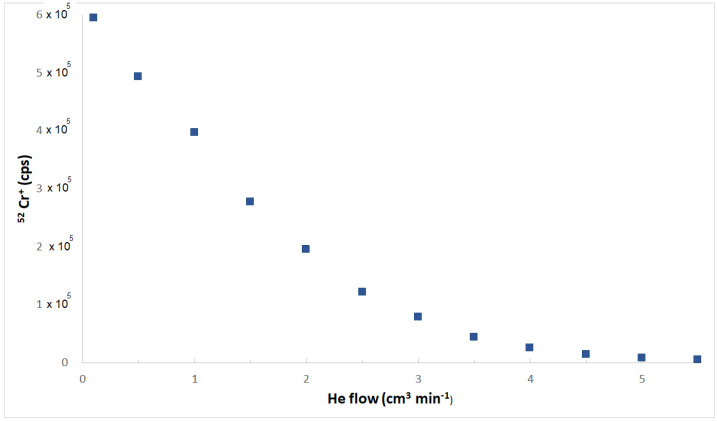
Dependence of the ionic signal of the ^52^Cr^+^ ion at variations of the He flow.

Whereas the elemental settings used for the determination of Al, As, Cd, Cr, Cu, Fe, Hg, Mn, Mo, Ni, Pb, Se, Sb, Tl, and Zn were from methods previously assessed by this research group [55,56,57], slightly modified to optimize them towards this matrix, those used to quantify B, Ba, Be, Bi, Co, Li, Sn, and U were specifically devoted to e-liquids. In particular, the choice of quantification ion is extremely important for the overall reliability of the method. It should be the best compromise between the lowest LoD and the minimization of any possible interference. Be, Bi, and Co are monoisotopic, hence no alternative is possible. For elements showing a multiplicity of isotopes, the most abundant one provides generally the highest instrumental sensitivity, and this is a very appealing feature when very low concentrations must be measured. For this reason, ^11^B, ^138^Ba, ^7^Li, ^120^Sn, and ^238^U, respectively, were chosen for the quantification of these elements in this study. The interference from molecular ions (mainly from oxides, carbides, nitrides, hydrides, or Ar-based species formed in the plasma, but also from polyatomic ions that originated from elements contained in high amounts in the matrix) is one of the most meaningful causes of bias in ICP-MS measurements [58]. Its presence/absence for each analyte has been established based on the behavior of the ionic signal measured at the increasing of He flows. A change of the slope of the decreasing trend of the ionic signal measured on a real sample at the increasing of the He flow accounted for the presence of an interference by molecular ions [59]. For explanatory purposes, Figure 2 reports the behavior of the ionic signal of the ^52^Cr^+^ ion at variations of the He flow. This behavior is similar to those observed for the remaining elements determined in KED mode.

It is evident that the first linear tract of the curve (He flow up to 2 cm^3^ min^−1^) gives an account for the removal of the molecular ionic interference, likely due to the ^40^Ar^12^C^+^ ion, whereas the second linear tract of the curve (roughly parallel to the *x*-axis, He flows higher than 4 cm^3^ min^−1^) accounts for a slight reduction of signal of the elemental ion as a function of the increase of the He flow. The abscissa of the intersection among both linear plots provided the optimized He flow aimed to remove the interference, which has been observed for 14 elements out 23. Hence, while the number of ionic counts of B, Be, Bi, Li, Hg, Mo, Pb, Tl, and U was measured in normal mode, the bias from polyatomic ions has been minimized for the remaining elements, using He flows between 3 cm^3^ min^−1^ and 4 cm^3^ min^−1^.

#### 2.1.3. Quantification, Quality Assurance and Quality Control

**Figure 3:**
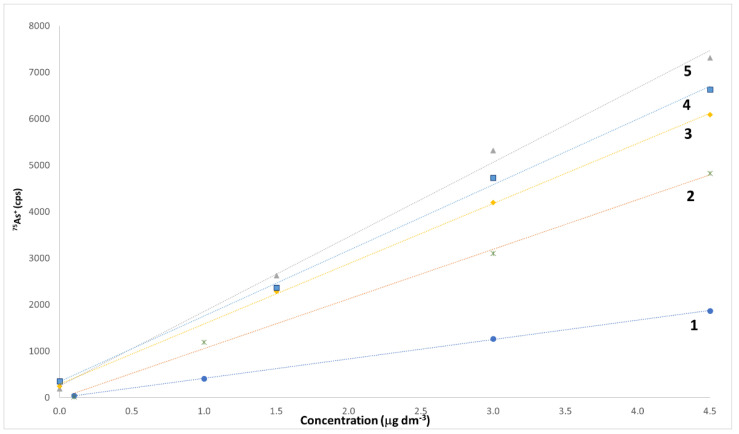
Linear calibration plots obtained using both external and internal calibration for the determination of As. Line 1, external calibration, As concentration in the range between 0.1 and 4.5 μg dm^−3^ in 2% (*v/v*) HNO_3_ in water; line 2, external calibration, As concentration in the range between 0.1 and 4.5 μg dm^−3^ in 2% (*v/v*) HNO_3_ in synthetic matrix; line 3, internal calibration for a tobacco flavor sample; line 4, internal calibration for a tonic flavor sample; line 5, internal calibration for a fruity flavor sample. In calibration lines 3–5, the amounts of As added to samples are of 113 pg, 226 pg, and 339 pg, respectively.

Both external and internal calibration approaches have been used in this study. External calibration (using either analyte standard solutions dissolved in water or in a synthetic matrix, henceforward called SM, formed by 54% of PG, 43% of VG, and 3% of water, respectively) has always been used for all pre-treatments chosen (dilution or microwave-assisted mineralization), but the ascertainment of a severe matrix effect in the quantification of almost all elements suggested the use of the internal calibration, accomplished by means of multiple additions of standard solutions. Figure 3 reports the linear calibration plots obtained using both external and internal calibration for the determination of As.

Constant and proportional bias due to matrix effects are well evident by the comparison of the behaviors of the two external calibration plots (obtained either on a 2% (*v/v*) HNO_3_ solution in water, line 1, or on a 2% (*v/v*) HNO_3_ solution in SM, line 2) and of the three internal calibration plots, obtained on tobacco, tonic, and fruity flavors, respectively. A similar behavior is also evident for the remaining elements quantified. It is well known that the internal calibration method has evident disadvantages with respect to the external calibration method. Firstly, it is cumbersome and quite time-consuming, since it requires the preparation of calibration curves for each individual sample and the relevant blanks [60]. In addition, it is characterized by a very high uncertainty, due to the widening of the confidence range of the internal calibration plot [61]. Conversely, its careful execution can almost suppress any matrix interferences, allowing the obtainment of a basically bias-free measurement [62]. A 10 μg dm^−3^ solution of Rh was used as an internal standard to compensate for any possible signal instability, while a washing cycle of at least 80 s was interposed between two consecutive samples to eliminate any possible memory effect. All data have been blank-corrected. In order to constantly monitor the overall level of accuracy of the method, one reagent blank every five samples was analyzed, whereas a standard solution containing 0.1 μg kg^−1^ of Be, Bi, Cd, Co, Tl, and U, 1 μg kg^−1^ of Li, Pb, Sb, and Se, 10 μg kg^−1^ of As, Al, Cu, and Mn, 50 μg kg^−1^ of B, Ba, Cr, and Hg, and 100 μg kg^−1^ of Fe, Mo, Ni, Sn, and Zn in SM was analyzed every ten samples. Each sample was analyzed in duplicate, and each piece of analytical data is the average of four replicated ICP-MS measurements.

### 2.2. Validation

Validation was performed in terms of Limit of Detection (LoD), Limit of Quantification (LoQ), and precision. Table 2 reports the relevant figures for all the parameters evaluated.

LoD and LoQ have been calculated according to Currie [63]. The standard deviation needed for the calculation of the LoD (and, hence, also for the LoQ) was calculated on the repeated measurement of 15 blanks. At last, for the elements previously measured in e-liquids, the data obtained are comparable with those reported in the literature [25,36,41,42]. Precision has been measured in terms of repeatability, twice quantifying the same real sample along the whole analytical process in the same analytical session by means of an internal calibration method (multiple standard addition). Quite high CV% values have been obtained for all parameters, ranging between 10% (for Al) and 100% (for Cu and Se). Since Se has always been found below its LoD for all samples of e-liquids, the precision was evaluated for this element by spiking the samples with the amount of Se standard solution needed to reach a final concentration in the samples slightly over the LoQ (i.e., 15 μg kg^−1^). Regardless, it is well known that, for the peculiar features of the standard addition method, the precision parameters measured that work in this way are much worse than those measured by quantifying by external calibration [61]. Trueness is usually measured by (i) analyzing a certified reference material (CRM), (ii) by comparison with an independent and validated analytical method, or, ultimately, (iii) with recovery tests, performed by spiking the sample with repeated amounts of pure analyte. Unfortunately, none of these methods can be used in this case. To the best of our knowledge, no CRM of e-liquids is commercially available, and the heavy matrix effect observed in this study, well substantiated in Figure 3, discourages using any apparently “similar” matrix. In addition, no independent and validated analytical method capable of measuring the concentration of 23 elements at concentrations no higher than a few μg dm^−3^ is currently available in our labs. Finally, the close similarities among the application of both the standard addition method and the recovery tests based on spiked samples suggest also discarding this option. In the attempt to obtain a tentative evaluation of the trueness of the method, a “synthetic e-liquid” was prepared, where known amounts of each analyte were added to the SM solution to reach a concentration close to the average value measured for the real samples. For the analytes that were not frequently quantified, their final concentration in the synthetic e-liquid was the LoQ. Multiple spiking tests, performed on this sample, allowed the obtainment of recoveries between 81% (for Cr) and 118% (for Zn), exhibiting hence a negligible level of bias if compared to the very low level of concentration measured.

### 2.3. Concentration of 23 Elements in 37 e-Liquids

The data relative to the determination of the amount of 23 elements in 37 e-liquids belonging to three main flavors (i.e., fruity, tobacco, and tonic) are summarized, in terms of both mean and ranges, in Table 3.

The data reported in Table 3 show that all e-liquids considered exhibit a very low amount of the evaluated analytes, being only ca. 0.6 mg kg^−1^, the sum of their average concentrations measured in this study. These amounts are comparable with the literature data [25,36,41] and confirm that the amount of potentially toxic elements contained in e-devices is orders of magnitude less than in traditional cigarettes [44,45]. Among all elements considered, only Se has always been found below its LoD in all samples analyzed. This is not surprising, knowing the scarce sensitivity of Se in terms of counts per second as a function of its concentration [59]. In addition, the mineralization process implies a dilution of 50 times of the sample, hence the effective LoD for mineralized solutions is 92 ng dm^−3^, i.e., quite close to the instrumental detection limit [64]. As far as the remaining elements are concerned, only six elements out of 36 (i.e., As, Co, Cr, Fe, Li, and Mn) exhibit a mean concentration higher than the LoQ. Only three elements, i.e., As, Cr, and Li, are always quantified (100% of the samples), but none of them seem to represent an effective health threat for vapers. Indeed, the highest amount measured for these elements is 11 μg kg^−1^ for As and 40 μg kg^−1^ for Cr. Only for the sake of comparison, the limits posed by the EU guidelines for these elements in water intended for human use are 10 μg kg^−1^ for As and 50 μg kg^−1^ for Cr, respectively [65], whereas the amount of daily intake for humans of water is, obviously, several orders of magnitude higher than that of e-liquids. Additionally, Li is less abundant than As and Cr, and its average concentration is only 1.9 μg kg^−1^. The average concentration of the elements most frequently quantified are, in decreasing percentage, Co (average concentration <0.34 μg kg^−1^, quantified in the 84% of samples), Zn (<109 μg kg^−1^, 76%), Mn (<10 μg kg^−1^, 73%), Sb (<2.9 μg kg^−1^, 73%), and Fe (<308 μg kg^−1^, 65%). Among the remaining toxic elements, only Sn and Ba have been quantified in over 25% of the samples, where the remaining elements, i.e., Be, Cd, Hg, Pb, Tl, and U, had seldom reached the relevant LoQs.

Table 4 reports a comparison between the data (mean and range) here obtained and those reported in the literature. To favor a reliable comparison among data obtained in different studies, only those obtained by using ICP-MS methods have been considered in this table.

Among the literature contributions considered in Table 4, V is the only element not analyzed in this study, but it previously was quantified by Beauval et al. [25,41]. Conversely, the amounts of B, Ba, Bi, Li, Mo, and U in e-liquids were measured for the first time in this study. Another element never quantified in previous studies, i.e., Se, was found to be below its LoD in this research. The amounts of elements analyzed in this study are in good agreement with results reported using validated methods for the determination of several elements in a statistically significant number of samples [25,36,41]. The most important differences regard the most abundant (and the most interfered) elements, such as Al, Fe, and Zn, which were frequently present in higher concentrations in this study. On the other hand, the amounts of another “critical” element, such as Ni, are higher in the literature studies than in this research. For elements such as As, Cd, Ni, and Pb, data here reported are in fair agreement with those reported by Song et al. [43], even if the validation of the method used in that contribution was not reported in the paper. On the whole, and with only very rare exceptions, such as the quite high amounts of Sb in a few of the e-liquids measured by Beauval et al. [41], the amounts of toxic elements found in e-liquids in this, as well as in previous studies [25,36,37,41,42,43], are coherent with a negligible health risk associated to its intake by vapers. The data measured here are several orders of magnitude below the worrying amounts measured by Hess et al. [33] for Cd, Cu, Mn, Ni, and Pb in two of the most popular brands of e-liquids sold in the USA. On the other hand, these concentrations, albeit much higher than those measured in the cited literature studies, are within the amounts recommended by the AFNOR [52], i.e., 1 mg kg^−1^ for Cd and Hg, 3 mg kg^−1^ for As, 5 mg kg^−1^ for Sb, and 10 mg kg^−1^ for Pb.

**Figure 4:**
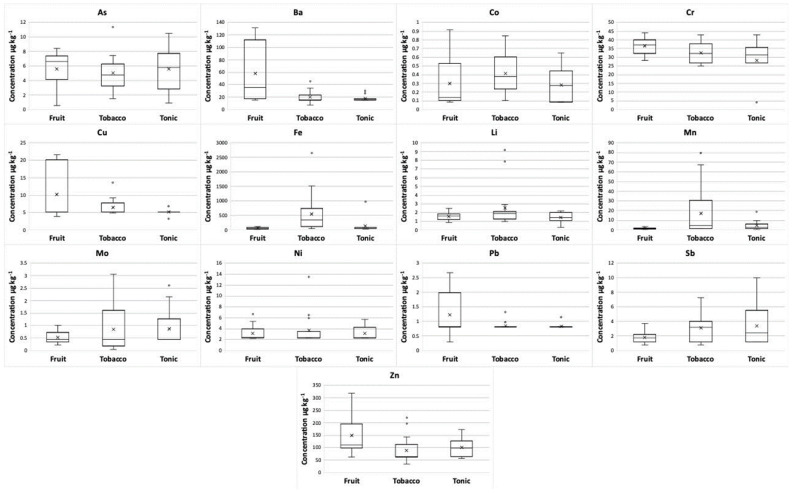
Box-whisker plots of the concentrations of As, Ba, Co, Cr, Cu, Fe, Li, Mn, Mo, Ni, Pb, Sb, and Zn in the three different flavor classes of e-liquids. The horizontal lines in the box represent the 25th percentile, the mean value, and the 75th percentile, respectively, and the interval between the ends of the whiskers represents the range. The “x” symbol is the median value, and the “_°_” symbol represents the outlier amounts.

Data reported also account for some differences among the three classes of flavors. Figure 4 shows the box-whisker plots for the concentrations of the most representative elements under exam in nine fruity, sixteen tobacco, and twelve tonic e-liquids. As a general behavior, tobacco flavors are the richest in the considered elements, whereas tonic flavors are the less abundant. In particular, tobacco flavors exhibit the highest amounts of Al, Bi, Cd, Fe, Hg, Li, Mn, Mo, and Ni, whereas the amounts of Al, Be, Bi, and Cd are always below the relevant LoDs in tonic flavors. Interestingly, fruity flavors show the highest amounts of some bivalent elements such as Ba, Be, Cu, Pb, Sn, and Zn.

### 2.4. Concentration of 23 Elements in PG, VG, Water, and Nicotine Used in the Composition of the e-Liquids

Table 5 reports the concentration of the 23 elements under exam in all the compounds used for the preparation of the e-liquids, with the only exclusion being of the concentrated flavor, always present in a 10% (*w/w*) amount in the final composition of the e-liquid.

Organic samples were analyzed using the same method developed for e-liquids, whereas water was analyzed using literature official methods [66,67]. The data reported in Table 5 give an account for an overall negligible contribution of toxic elements in the composition of the e-liquids. On the other hand, it is evident that the most abundant elements in e-liquids come from VG (i.e., As, Cr, Cu, Li, and Zn) and PG (i.e., B, Cr, Fe, and Zn). Furthermore, the water is quite rich in B, whereas nicotine (always absent in all the samples analyzed, see Section 3.1) is very rich in Al (5 mg kg^−^^1^). Moreover, the amounts of Cr, Zn, and Sb are significant. Since the concentration of Cr is relatively elevated in both VG and PG, it is likely that the high amount of this element measured in the aerosols can depend not only on the metal constituents of the e-cigarette, but also on the aliquot of Cr present in the organic solvents in the e-liquids. Hence, a reduction of the elemental concentrations of the main trace elements contained in the e-liquids would be an effective tool to further reduce the amount of oligoelements in them.

### 2.5. Principal Components Analysis

**Figure 5:**
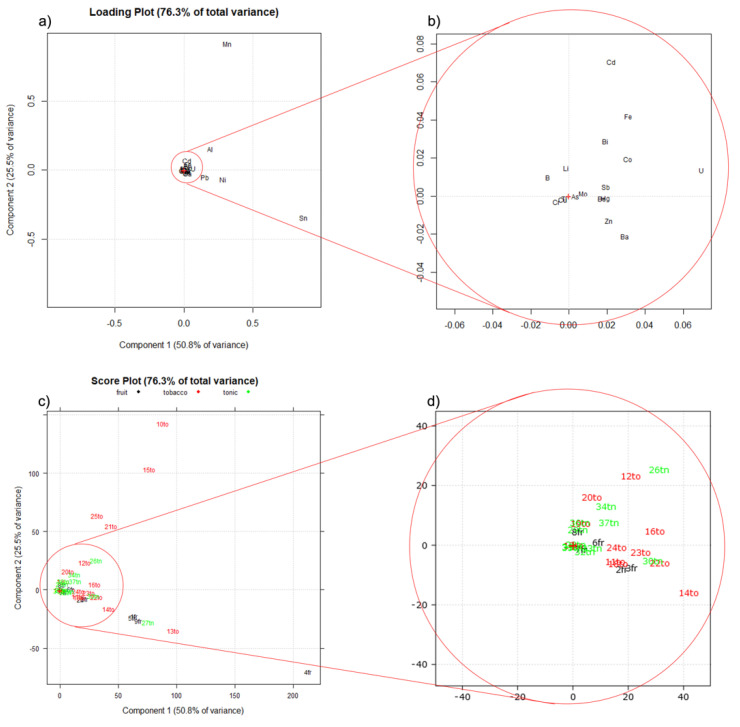
PCA performed on the same dataset processed and normalized, for each analyte, with respect to the relevant concentration measured in the synthetic matrix (SM). (**a**) Loading plot; (**b**) zoomed view of the loading plot; (**c**) score plot; (**d**) zoomed view of the score plot; to: tobacco flavored samples; fr: fruity-flavored samples; tn: tonic-flavored samples.

In order to verify which elements were deriving either from the constituents or from the flavors, the whole dataset consisting of 37 samples of e-liquids and 22 elements (Se was always found below its LoD and thus excluded) was normalized with respect to the concentration of each element measured in the SM (i.e., in the absence of any contribution given by any flavor), obtaining a dataset expressed in terms of standard deviation referred to in the SM concentration. A principal components analysis (PCA) was carried out on the normalized dataset. Figure 5 shows the loading plot (a), with its zoomed view (b), and the score plot (c), with its zoomed view (d).

The two principal components explain 76% of the total variance. In the loading plot (Figure 5a,b), 17 out the 22 elements quantified are crowded around the center of the plot, giving no contribution to the explained variance, whereas elements such as Al, Pb, Ni, and mainly Mn and Sn are responsible for the variability expressed by the first two components. In the score plot, most of the samples are grouped in the origin of the two PC axes, with only a few samples highly differing from the others. To interpret the obtained PCA, it must be kept in mind that data were normalized with respect to the concentrations of the elements in the SM; this means that the samples grouping around the origin of the axes in the score plot (Figure 5c,d) show no variability with respect to the SM, whereas samples distant from the origin are those that mostly differ from the SM. This difference is due to a few elements (Mn, Sn and, to a minor extent, Al, Pb, and Ni are responsible for the variability seen in the plots). This means that the tobacco samples 10to, 15to, 21to, and 25to, described by high positive score values of PC2, are the richest in Al and, in particular, Mn, with respect to the SM. On the other hand, the samples 4fr, 13to, 27tn, 5fr, 9fr, and 1fr, described by high positive score values of PC1 and negative score values of PC2, are richer in Sn, Ni, and Pb, with respect to the SM.

Looking at the zoomed view of the plots (Figure 5b,d), it is interesting to observe that positive scores of PC1 are correlated by the increase of the amount of each analyte in the e-liquid, with respect to the amount present in the SM. The almost complete absence of elements and samples described by negative scores of PC1 is, in our opinion, a clear marker of the overall reliability of the data here obtained. Summarizing, Sn, Mn, and—in decreasing amounts—Ni, Al, and Pb, are elements that mainly originated from the flavors, whereas the remaining elements are derived mainly from the constant compounds of the e-liquids, i.e., PG, VG, and water. In addition, it is interesting to observe that tonic e-liquids are the category closest to the matrix concentration, whereas fruity and tobacco e-liquids are the most scattered samples.

## 3. Materials and Methods

### 3.1. Samples and Reagents

The 37 samples of e-liquids were provided by a local producer. Eight of them were fruity-flavored, twelve were tonic-flavored, and seventeen were tobacco-flavored. Table 6 reports the composition of each class of e-liquids.

The same producer also provided the pure compounds (i.e., propylene glycol, glycerin, and water) that were added to the concentrated flavor to produce the samples analyzed. Albeit they were all nicotine-free, the producer also provided a sample of pure nicotine to evaluate its contribution to the overall amount of trace elements in the nicotine-added e-liquids.

HNO_3_ 69% (NORMATON for ultra-trace analysis, VWR, Milan, Italy) and type I water (MilliQ plus System, Millipore, Vimodrone, Italy) were used in all the phases of the study. Triton^TM^ X-100 aqueous solution, 10%(*w/v*) was from Merck, Milan, Italy. ICP-MS elemental standards of Al, As, B, Ba, Be, Bi, Cd, Co, Cr, Cu, Fe, Hg, Li, Mn, Mo, Ni, Pb, Sb, Sn, Tl, U, and Zn (1000 mg dm^−3^ each) were purchased from LabKings (Hilversum, The Netherlands). The ICP-MS setup solution (a 1% *v/v* aqueous solution of HNO_3_ containing 1 μg dm^−3^ each of Be, Ce, Fe, In, Li, Mg, Pb, and U), the ICP-MS KED setup solution (a 1% (*v/v*) aqueous solution of HNO_3_ containing 10 μg dm^−3^ of Co and 1 μg dm^−^^3^ of Ce), and the internal standard solution (10 μg dm^−^^3^ of Rh in a 1% *v/v* aqueous solution of HNO_3_) were all from Perkin Elmer, Milan, Italy. All the samples were filtered before the analysis using a polypropylene filter (pore diameter: 0.22 µm) from VWR, Milan, Italy.

### 3.2. Instrumentation

The samples were mineralized using a microwave SRC system (UltraWave™, Milestone, Sorisole, Italy). The elemental determination was performed using a NexION 300X ICP-MS spectrometer (Perkin Elmer, Milan, Italy) equipped with a nebulization system composed of a glass concentric nebulizer, a glass cyclonic spray chamber, an autosampler model S10, and a KED collision cell. All the elements were determined simultaneously, and the information about the analytes and operational conditions has already been reported in Table 1.

### 3.3. Statistical Analysis

Principal components analysis (PCA) was performed by means of the R-based software Chemometric Agile Tool (CAT) developed by the Italian group of chemometrics [68].

## 4. Conclusions

The perceived need in the literature for sensitive, reliable, and validated ICP-MS methods to quantify a wide range of both toxic elements and oligoelements in e-liquids is the main reason for accomplishing this study. Hence, an original method capable of determining the concentration of 23 elements in such a matrix has been developed and validated in terms of LoD, LoQ, precision, and trueness. The need to circumvent the very strong matrix effect has required the introduction of significant changes with respect to the methods described in the literature. Namely, the sample pre-treatment step has been accomplished by microwave-assisted mineralization. Moreover, the ICP-MS instrumental parameters have been optimized to enhance the sensitivity and reduce the spectral interference by the polyatomic ions. Finally, the quantification, performed by means of an internal calibration with three additions of standards, has allowed for the obtainment of accurate data. The method has been applied to 37 different e-liquids of three different classes of flavors (i.e., fruity, tobacco, and tonic). The average concentration of all analytes is aligned with the lowest amounts measured in the literature, confirming hence that, also in this case, the potential health risk related to the amount of toxic elements in e-liquids is orders of magnitude less than that measured for traditional cigarettes. Almost all toxic elements have been found below an amount lower than a few μg kg^−1^ and, very often, they were below the relevant LoQ. The data obtained also give an account for the slight differences among the classes of flavors. Tobacco and tonic flavors exhibit the highest and the lowest amounts of elements, respectively. The results of the PCA showed that the multivariate approach allowed easy differentiation of the elements derived from constituents and those from added flavors. For this reason, this approach may be generalized in order to minimize the amount of each element in the final e-liquids. In conclusion, e-liquids seem to be scarcely involved as potential sources of toxic elements inhaled by vapers. Hence, further investigations are needed to ascertain the influence of the conditions of use for e-cigarettes on the dragging in the vapor phase for the elements of potential toxicity in humans.

## Figures and Tables

**Table 1:** Instrumental parameters and elemental settings used for the ICP-MS determination of 23 toxic elements and oligoelements in e-liquids.

**RF power generator (W)**	1300	**KED ^a^ mode cell entrance voltage (V)**		−8.0
**Ar plasma flow (dm^3^ min^−1^)**	18.0	**KED mode cell exit voltage (V)**		−25.0
**Ar auxiliary flow (dm^3^ min^−1^)**	1.20	**Resolution (Da)**		0.7
**Ar nebulizer flow (dm^3^ min^−1^)**	0.91	**Scan mode**		Peak hopping
**Nebulizer**	Meinhard^®^, glass	**Detector mode**		Dual
**Spray chamber**	Cyclonic, glass	**Dwell time (ms)**		50
**Skimmer and sampling cones**	Nickel	**Number of points per peak**		3
**Sampling depth (mm)**	0	**Acquisition time (s)**		6
**Deflector voltage (V)**	−8.00	**Acquisition dead time (ns)**		35
**Analog stage voltage (V)**	−1750	**KED gas**		Helium, 99.999%
**Pulse stage voltage (V)**	+1350	**Masses of optimization**		^7^Li, ^89^Y and ^205^Tl
**Quantification Ion** **(% abundance)**	**Interferents**	**Analyzing Mode**	**He Flow** **(cm^3^ min^−1^)**	**Correction** **Equation**
^27^ Al^+^ (100)	^11^B^16^O^+^; ^13^C^14^N^+^; ^11^Be^16^O^+^; ^26^Mg^1^H^+^; ^12^C ^15^N^+^; ^54^Cr^2+^; ^54^Fe^2+^	KED	3.5	none
^75^ As^+^ (100)	^40^Ar^35^Cl^+^; ^59^Co^16^O^+^; ^39^K^36^Ar^+^; ^63^Cu^12^C^+^; ^40^Ca^35^Cl^+^; ^58^Ni^16^O^1^H^+^	KED	3.0	none
^11^ B^+^ (80.1)	none	Normal		none
^138^ Ba^+^ (71.7)	^40^Ar_2_^58^Ni^+^; ^138^La^+^; ^122^Sn^16^O^+^; ^137^Ba^1^H^+^; ^121^Sb^16^O^1^H^+^	KED	4.0	−0.000901 × ^139^La −0.002838 × ^140^Ce
^9^ Be^+^ (100)	none	Normal		none
^209^ Bi^+^ (100)	none	Normal		none
^111^ Cd^+^ (12.80)	^95^Mo^16^O^+^; ^97^Mo^14^N^+^; ^79^Br^16^O_2_^+^; ^94^Zr^16^O^1^H^+^; ^71^Ga^40^Ar^+^	KED	4.0	none
^59^ Co^+^(100)	^43^Ca^16^O^+^; ^42^Ca^16^O^1^H^+^; ^24^Mg^35^Cl^+^; ^40^Ar^18^O^1^H^+^; ^118^Sn^2+^; ^27^Al^16^O_2_^+^; ^58^Ni^1^H^+^; ^24^Mg^35^Cl^+^	KED	3.5	none
^52^ Cr^+^ (83.79)	^40^Ar^12^C^+^; ^36^Ar^16^O^+^; ^1^H^35^Cl^16^O^+^; ^104^Pd^2+^; ^51^V^1^H^+^; ^40^Ca^12^C^+^; ^38^Ar^14^N^+^	KED	3.0	none
^63^ Cu^+^ (69.17)	^40^Ar^23^Na^+^; ^31^P^16^O_2_^+^; ^47^Ti^16^O^+^; ^28^Si^35^Cl^+^; ^51^V^12^C^+^	KED	4.0	none
^57^ Fe^+^ (2.12)	^40^Ar^16^O^1^H^+^; ^40^Ca^16^O^1^H^+^; ^40^K^16^O^1^H^+^	KED	3.0	none
^7^ Li^+^ (92.50)	none	Normal		none
^202^ Hg^+^ (22.86)	^186^W^16^O^+^	Normal		none
^55^ Mn^+^ (100)	^40^Ar^14^N^1^H^+^; ^37^Cl^18^O^+^; ^39^K^16^O^+^	KED	3.0	none
^98^ Mo^+^ (24.13)	^98^Ru^+^; ^81^Br^17^O^+^; ^40^K_2_^18^O^+^; ^58^Ni^40^Ar^+^; ^63^Cu^35^Cl^+^	Normal		−0.10961 × ^101^Ru
^60^ Ni^+^ (26.22)	^44^Ca^16^O^+^; ^43^Ca^16^O^1^H^+^; ^23^Na^37^Cl^+^; ^25^Mg^35^Cl^+^; ^28^Si^16^O_2_^+^	KED	3.5	none
^208^ Pb^+^ (52.40)	none	Normal		none
^121^ Sb^+^ (57.21)	^107^Ag^14^N^+^; ^109^Ag^12^C^+^; ^105^Pd^16^O^+^; ^81^Br^40^Ar^+^; ^120^Sn^1^H^+^	KED	3.5	none
^82^ Se^+^ (8.73)	^82^Kr^+^; ^81^Br^1^H^+^; ^66^Zn^16^O^+^; ^68^Zn^14^N^+^; ^164^Dy^2+^; ^65^Cu^16^O^1^H^+^	KED	3.5	−0.00783 × ^83^Kr
^120^ Sn^+^ (32.58)	^39^K^81^Br^+^; ^80^Se^40^Ar^+^; ^104^Pd^16^O^+^; ^104^Ru^16^O^+^	KED	3.5	none
^205^ Tl^+^ (70.26)	^189^Os^16^O^+^	Normal		none
^238^ U^+^ (99.3)	none	Normal		none
^66^Zn^+^ (27.90)	^50^Ti^16^O^+^; ^34^S^16^O_2_^+^; ^132^Ba^2+^; ^50^Cr^16^O^+^; ^65^Cu^1^H^+^; ^26^Mg^40^Ar^+^; ^31^P^35^Cl^+^; ^52^Cr^14^N^+^	KED	3.0	none

^a^ Kinetic Energy Discrimination, KED.

**Table 2:** Validation parameters for the ICP-MS determination of the total amount of 23 toxic elements and oligoelements in e-liquids.

Element	LoD (μg kg^−1^)	LoQ (μg kg^−1^)	Repeatability (CV%)	Element	LoD (μg kg^−1^)	LoQ (μg kg^−1^)	Repeatability (CV%)
Al	26	84	10	Li	0.37	1.2	40
As	0.51	1.7	40	Mn	1.6	5.1	40
B	37	120	60	Mo	0.45	1.5	70
Ba	15	50	30	Ni	2.3	7.4	60
Be	0.057	0.19	70	Pb	0.80	2.7	40
Bi	0.089	0.29	80	Sb	1.1	3.7	50
Cd	0.12	0.39	90	Se	4.6	15	100
Co	0.089	0.29	60	Sn	0.24	0.78	30
Cr	4.2	14	70	Tl	0.055	0.18	50
Cu	5.2	17	100	U	0.21	0.69	30
Fe	53	180	90	Zn	62	200	30
Hg	4.5	15	90				

**Table 3:** Mean amounts, ranges (both in μg kg^−^^1^), and percentage of quantified samples (C > LoQ) in the determination of 23 toxic elements and oligoelements in 37 different e-liquids.

Elements	All Flavors (*n* = 37) (Mean; Range; % Samples > LoQ)		Fruity Flavors (*n* = 9) (Mean; Range; % Samples > LoQ)		Tobacco Flavors (*n* = 16) (Mean; Range; % Samples > LoQ)		Tonic Flavors (*n* = 12) (Mean; Range; % Samples > LoQ)	
Al	<33; *<26*–160	8%	<35; *<26*–110	11%	<37; *<26*–160	13%	*<26*; *<26*; *<26*	0%
As	5; 0.6–11	100%	6; 0.6–8	100%	5; 1.5–11	100%	6; 0.8–10	100%
B	<61; *<37*–140	54%	<50;*<37*–100	33%	<60; *<37*–100	56%	<70; *<37*–140	67%
Ba	<27; *<15*–130	62%	<55; *<15*–130	78%	<20; *<15*–45	63%	<18; *<15*–30	50%
Be	<0.06; *<0.057*–0.12	14%	<0.07; *<0.057*–0.12	33%	<0.058; *<0.057*–0.06	13%	*<0.057*; *<0.057*–*<0.057*	0%
Bi	<0.11; *<0.089*–0.3	27%	<0.09; *<0.089*–0.1	11%	<0.14; *<0.089*–0.3	56%	*<0.089; <0.089–<0.089*	0%
Cd	<0.14; *<0.12*–1	5%	*<0.12*; *<0.12*–*<0.12*	*0%*	<0.18; *<0.12*–1	6%	*<0.12; <0.12*–0.13	8%
Co	<0.34; *<0.089*–0.9	84%	<0.3; *<0.089*–0.9	78%	<0.4; 0.1–0.8	100%	<0.3; *<0.089*–0.6	67%
Cr	34; 20–40	100%	38; 30–40	100%	34; 30–40	100%	32; 20–40	100%
Cu	<7.1; *<5.2*–20	41%	<10; *<5.2*–20	56%	<7; *<5.2*– 14	50%	<5.4; *<5.2*–7	17%
Fe	<308; *<53*–3000	65%	<66; *<53*–100	56%	<570; *<53*–3000	88%	<140; *<53*–1000	42%
Hg	<5; *<4.5*–14	8%	<5; *<4.5*–10	11%	<5; *<4.5*–14	6%	<5; *<4.5*–5	8%
Li	1.9; 0.7–9	100%	1.5; 0.8–2	100%	2.5; 0.9–9	100%	1.5; 0.7–2.2	100%
Mn	<10; *<1.6*– 80	73%	<2; *<1.6*–4	67%	<18; *<1.6*–80	69%	<5; *<1.6*–20	83%
Mo	<0.8; *<0.45*–3	57%	<0.6; *<0.45*–1	44%	<1; *<0.45*–3	81%	<0.7; *<0.45*–2	33%
Ni	<3.5; *<2.3*–14	46%	<3.4; *<2.3*–7	67%	<3.8; *<2.3*–14	44%	<3.2; *<2.3*–6	33%
Pb	<1.0; *<0.8*–3	19%	<1.4; *<0.8*–3	44%	<0.84; *<0.8*–1.3	13%	<0.81; *<0.8*–1	8%
Sb	<2.9; *<1.1*–10	73%	<1.8; *<1.1*–4	78%	<3.1; *<1.1*–7	75%	<3.4; *<1.1*–10	67%
Se	*<4.6*; *<4.6*–*<4.6*	*0%*	*<4.6*; *<4.6*–*<4.6*	*0%*	*<4.6*; *<4.6*–*<4.6*	*0%*	*<4.6*; *<4.6*–*<4.6*	0%
Sn	<0.6; *<0.24*–4	65%	<1; *<0.24*–4	67%	<0.5; *<0.24*–1.6	81%	<0.37; *<0.24*–1.5	42%
Tl	<0.07; *<0.055*–0.16	46%	<0.07; *<0.055*–0.16	22%	<0.06; *<0.055*–0.15	38%	<0.08; *<0.055*–0.15	75%
U	<0.29; *<0.21*–0.7	41%	<0.29; *<0.21*–0.6	44%	<0.31; *<0.21*–0.6	44%	<0.26; *<0.21*–0.7	33%
Zn	<109; *<62*–300	76%	<150; *<62*–300	89%	<90; *<62*–220	63%	<100; *<62*– 170	83%

Each sample has been analyzed twice. In italics: data below the LoD; in underlined: data below the LoQ. All average data have been rounded after calculation. The average data prefixed with the sign “<“ have been calculated based on at least one concentration that has been found below the corresponding LoD.

**Table 4:** Average amounts and ranges (in μg kg^−1^) of elements of health concern in e-liquids. Concentration was measured by means of ICP-MS methods.

Elements	Ref. [25] ^a^ (*n* = 6)	Ref. [33] ^b^ (*n* = 5)	Ref. [36] ^c^ (*n* = 56)	Ref. [37] ^d^ (*n* = 1)	Ref. [41] ^a^ (*n* = 27)	Ref. [42] ^e^ (*n* = 2)	Ref. [43] ^f^ (*n* = 3)	This Study (*n* = 37)
Al	12; 10–15		50.3; 46.22–59.6	7.7 ± 0.5	12.9; 8.82–30.7			<33; *<26*–160
As	1.2; <1–1.5			0.08 ± 0.04	1.57; <1–3.42	<430	2.18; 0.83–3.04	5; 0.6–11
B								<61; *<37*–140
Ba								<27; *<15*–130
Be	<0.1				<0.1			<0.06; *<0.057*–0.12
Bi								<0.11; *<0.089*–0.3
Cd	<0.4	43.5; 0.137–755	<0.1	<0.01	<0.4	<220	0.54; <0.25–1.28	<0.14; *<0.12*–1
Co	0.15; <0.1–0.27				0.262; <0.1–0.884			<0.34; *<0.089*–0.9
Cr	5.2; 4.1–7.7	669; 41.5–16900	12; 12–14.26		7.16; 4.08–11.5			34; 20–40
Cu	23; <20–32		5.14; <1.0–16.1	<0.01	27.0; <20–30.6			<7.1; *<5.2*–20
Fe			66.5; 48.74–130.9	4.1 ± 0.2				<308; *<53*–3000
Hg	<4				4.38; <4–4.54			<5; *<4.5*–14
Li								1.9; 0.7–9
Mn	2.1; <1.6–3.3	1627; 11.8–31500	1.09; <1.0–2.74	0.159 ± 0.006	3.99; <1.6–8.42			<10; *<1.6*– 80
Mo								<0.8; *<0.45*–3
Ni	<16	7613; 13.7–72700	7.33; 5.30–47.4	0.161 ± 0.007	<16		3.43; 1.42–5.11	<3.5; *<2.3*–14
Pb	<1	444; 3.17–4870	0.476; 0.243–1.05	<0.01	<1		12.28; <0.25–23.49	<1.0; *<0.8*–3
Sb	1.6; 1.2–1.5		1.0; 1.0–1.219		7.21; 0.400–214			<2.9; *<1.1*–10
Se								*<4.6; <4.6–<4.6*
Sn			1.53; 0.689–3.75					<0.6; *<0.24*–4
Tl	<0.1				<0.1			<0.07; *<0.055*–0.16
V	0.45; <0.4–0.64				0.602; <0.4–1.36			-
U								<0.29; *<0.21*–0.7
Zn	<200		18.2; 11.94–28.2	0.51 ± 0.03	418; <200–510			<109; *<62*–300

In italics: data below the LoD; in underlined: data below the LoQ. ^a^ Solvents: PG < 65%, VG < 35%, samples with (16 mg cm^−3^) or without nicotine. ^b^ E-liquids were popular brands sold in the USA. An interlaboratory trial confirmed these data. ^c^ Solvents: PG, 70% VG, 30%. Data are relative to median, where the range is within the 25th and the 75th percentile. Data reported in this column are the sum of those reported in Table 2 of the paper and the amount of the relevant blanks reported in Table S1 of the supplementary material. Data are from only one measurement for each sample. Trueness has been evaluated through an interlaboratory trial and by analysis of a NIST SRM^®®^ 1640a (Trace Elements in Natural Water). ^d^ E-liquid was 1:100 diluted with HNO_3_ 1% in water before ICP-MS analysis. All trace metal analyses were performed as a contracted service. No details were provided on the validation of the ICP-MS method. ^e^ MarkTen^®®^ Menthol and Classic e-liquids, both containing 1.5% nicotine. No details were provided on the validation of the ICP-MS method. ^f^ No details were provided on the nature and validation of the ICP-MS determination.

**Table 5:** Mean amounts (in μg kg^−1^± SD) of 23 toxic elements and oligoelements in VG, PG, water, and nicotine used for the preparation of the e-liquids. *n* = 3.

Element	VG	PG	Water	Nicotine
Al	*<26*	*<26*	*<5*	5000 ± 1000
As	7 ± 2	2.6 ± 0.6	0.020 ± 0.005	0.8 ± 0.2
B	*<37*	110 ± 10	470 ± 70	*<37*
Ba	*<15*	*<15*	0.18 ± 0.02	*<15*
Be	*<0.057*	*<0.057*	*<0.01*	*<0.057*
Bi	*<0.089*	*<0.089*	*<0.005*	*<0.089*
Cd	*<0.12*	*<0.12*	0.04 ± 0.02	*<0.12*
Co	0.2 ± 0.1	*<0.089*	*<0.005*	*<0.089*
Cr	56 ± 7	49 ± 6	*<0.1*	59 ± 8
Cu	22 ± 4	*<5.2*	*<0.1*	*<5.2*
Fe	0.6 ± 0.1	100 ± 20	*<1*	0.6 ± 0.1
Hg	*<4.5*	*<4.5*	*<0.005*	*<4.5*
Li	6 ± 1	*<0.37*	0.374 ± 0.001	<1.2
Mn	*<1.6*	*<1.6*	2.8 ± 0.2	11 ± 2
Mo	*<0.45*	*<0.45*	0.8 ± 0.2	*<0.45*
Ni	*<2.3*	*<2.3*	2.5 ± 0.6	*<2.3*
Pb	*<0.80*	*<0.80*	1.6 ± 0.1	*<0.80*
Sb	<3.7	*<1.1*	0.67 ± 0.01	120 ± 20
Se	*<4.6*	*<4.6*	0.035 ± 0.015	*<4.6*
Sn	*<0.24*	*<0.24*	*<0.01*	*<0.24*
Tl	<0.18	*<0.055*	*<0.001*	*<0.055*
U	*<0.21*	*<0.21*	*<0.003*	*<0.21*
Zn	180 ± 40	41 ± 9	5 ± 1	160 ± 30

VG: vegetal glycerin; PG: propylene glycol; SD: standard deviation; in italics: amounts below the LoD; underlined: amounts below the LoQ.

**Table 6:** Composition of the three classes of e-liquids.

e-Liquid Flavor Class	PG (%)	VG (%)	Concentrated Flavor (%)	Water (%)
Fruity	50	40	8	2
Tobacco	50	40	6	4
Tonic	50	40	7	3

## Supplementary Materials

The following table is available online. Table S1. Elemental concentration of toxic elements and oligoelements in 37 different e-liquids.

## Sample Availability

Samples of the compounds are not available from the authors but may be requested from the New Flavours company.
