# Supplementary material for: ICP-MS Determination of 23 Elements of Potential Health Concern in Liquids of e-Cigarettes. Method Development, Validation, and Application to 37 Real Samples
Source: Molecules. 2021 Nov 4;26(21):6680. doi: 10.3390/molecules26216680 (PMC8588553; doi:10.3390/molecules26216680)
Supplement: Supplementary file 1 [file molecules-26-06680-s001.zip › molecules-1440234-supplementary.pdf]

Table S1. Elemental concentration of toxic elements and oligoelements in 37 different e-liquids.

Flavor	ID	Commercial name	Al	As	B	Ba	Be	Bi	Cd	Co	Cr	Cu	Fe	Hg	Li	Mn	Mo	Ni	Pb	Sb	Se	Sn	Tl	U	Zn
Fruit	1fr	Bananita	*< 26*	8 ± 3	*< 37*	110 ± 30	*< 0.057*	*< 0.089*	*< 0.12*	0.1 ± 0.1	40 ± 30	20 ± 20	100 ± 80	*< 4.5*	1.9 ± 0.7	1.6 ± 0.6	*< 0.45*	3 ± 2	3 ± 1	2 ± 1	*< 4.6*	1.0 ± 0.3	*< 0.055*	0.3 ± 0.1	200 ± 60
Fruit	2fr	Coconut	*< 26*	7 ± 3	*< 37*	30 ± 10	*< 0.057*	*< 0.089*	*< 0.12*	0.1 ± 0.1	40 ± 30	6 ± 4	*< 53*	10 ± 10	0.8 ± 0.3	1.6 ± 0.6	*< 0.45*	*< 2.3*	*< 0.8*	4 ± 2	*< 4.6*	0.4 ± 0.1	0.16 ± 0.8	0.4 ± 0.1	*< 62*
Fruit	3fr	Fruit Mix	*< 26*	5 ± 2	*< 37*	*< 15*	*< 0.057*	*< 0.089*	*< 0.12*	0.2 ± 0.1	30 ± 20	*< 5.2*	60 ± 60	*< 4.5*	1.6 ± 0.6	1.6 ± 0.6	0.5 ± 0.4	3 ± 2	1.3 ± 0.5	1.3 ± 0.7	*< 4.6*	0.4 ± 0.1	*< 0.055*	0.3 ± 0.1	100 ± 30
Fruit	4fr	Mangos	110 ± 10	7 ± 3	*< 37*	130 ± 40	0.10 ± 0.07	0.1 ± 0.2	*< 0.12*	0.8 ± 0.5	30 ± 20	*< 5.2*	100 ± 70	*< 4.5*	1.3 ± 0.6	*< 1.6*	1.0 ± 0.7	7 ± 4	*< 0.8*	2 ± 1	*< 4.6*	4 ± 1	0.06 ± 0.03	0.6 ± 0.2	300 ± 100
Fruit	5fr	Mela Green	*< 26*	7 ± 3	*< 37*	110 ± 30	*< 0.057*	*< 0.089*	*< 0.12*	*< 0.089*	40 ± 30	20 ± 20	*< 53*	*< 4.5*	1.9 ± 0.7	*< 1.6*	*< 0.45*	3 ± 2	3 ± 1	2 ± 1	*< 4.6*	1.0 ± 0.3	*< 0.055*	*< 0.21*	200 ± 60
Fruit	6fr	Orange	*< 26*	7 ± 3	90 ± 50	40 ± 10	*< 0.057*	*< 0.089*	*< 0.12*	0.9 ± 0.6	40 ± 30	*< 5.2*	*< 53*	*< 4.5*	1.7 ± 0.6	3 ± 1	*< 0.45*	5 ± 3	1.3 ± 0.5	1.7 ± 0.9	*< 4.6*	*< 0.24*	*< 0.055*	*< 0.21*	100 ± 30
Fruit	7fr	Passion Fruit	*< 26*	4 ± 2	*< 37*	20 ± 10	*< 0.057*	*< 0.089*	*< 0.12*	*< 0.089*	40 ± 30	*< 5.2*	*< 53*	*< 4.5*	1.4 ± 0.6	*< 1.6*	1.0 ± 0.7	3 ± 2	*< 0.8*	1.3 ± 0.7	*< 4.6*	*< 0.24*	*< 0.055*	*< 0.21*	110 ± 30
Fruit	8fr	Pineapple	*< 26*	4 ± 2	100 ± 60	21 ± 6	0.12 ± 0.09	*< 0.089*	*< 0.12*	0.3 ± 0.3	40 ± 30	20 ± 20	60 ± 60	*< 4.5*	2 ± 1	4 ± 1	*< 0.45*	*< 2.3*	*< 0.8*	*< 1.1*	*< 4.6*	*< 0.24*	*< 0.055*	*< 0.21*	100 ± 30
Fruit	9fr	Sweet Red	*< 26*	0.6 ± 0.1	40 ± 20	*< 15*	0.07 ± 0.05	*< 0.089*	*< 0.12*	0.1 ± 0.1	40 ± 30	6 ± 5	60 ± 60	*< 4.5*	1.1 ± 0.4	1.6 ± 0.6	0.5 ± 0.4	*< 2.3*	*< 0.8*	*< 1.1*	*< 4.6*	1.5 ± 0.5	*< 0.055*	*< 0.21*	150 ± 50
Tobacco	10to	Concept Vapure	160 ± 20	3 ± 1	60 ± 40	*< 15*	0.06 ± 0.04	*< 0.089*	1.0 ± 0.8	0.6 ± 0.4	30 ± 20	9 ± 9	200 ± 100	*< 4.5*	8 ± 3	80 ± 30	0.5 ± 0.4	3 ± 2	*< 0.8*	*< 1.1*	*< 4.6*	0.6 ± 0.2	*< 0.055*	*< 0.21*	120 ± 40
Tobacco	11to	Gold Fire	*< 26*	4 ± 2	60 ± 30	15 ± 10	*< 0.057*	*< 0.089*	*< 0.12*	0.2 ± 0.1	30 ± 20	6 ± 4	*< 53*	*< 4.5*	1.8 ± 0.8	*< 1.6*	0.5 ± 0.4	3 ± 2	*< 0.8*	*< 1.1*	*< 4.6*	0.3 ± 0.1	*< 0.055*	0.5 ± 0.2	*< 62*
Tobacco	12to	Havana	*< 26*	4 ± 2	100 ± 60	15 ± 10	*< 0.057*	*< 0.089*	*< 0.12*	0.3 ± 0.2	30 ± 20	6 ± 6	3000 ± 2000	*< 4.5*	2.0 ± 0.8	16 ± 6	0.5 ± 0.4	4 ± 2	*< 0.8*	*< 1.1*	*< 4.6*	0.25 ± 0.07	*< 0.055*	*< 0.21*	*< 62*
Tobacco	13to	Hit Camel	*< 26*	3 ± 1	*< 37*	15 ± 5	*< 0.057*	*< 0.089*	*< 0.12*	0.2 ± 0.1	40 ± 30	8 ± 9	200 ± 100	*< 4.5*	0.9 ± 0.4	*< 1.6*	0.5 ± 0.4	14 ± 8	*< 0.8*	*< 1.1*	*< 4.6*	1.6 ± 0.5	*< 0.055*	*< 0.21*	*< 62*
Tobacco	14to	Hot Cigar	*< 26*	5 ± 2	80 ± 50	*< 15*	*< 0.057*	0.2 ± 0.1	*< 0.12*	0.5 ± 0.3	30 ± 20	*< 5.2*	800 ± 500	*< 4.5*	1.2 ± 0.5	*< 1.6*	0.9 ± 0.7	*< 2.3*	*< 0.8*	7 ± 4	*< 4.6*	1.0 ± 0.3	*< 0.055*	*< 0.21*	200 ± 60
Tobacco	15to	Latakia Easy	*< 26*	7 ± 3	80 ± 50	*< 15*	*< 0.057*	0.3 ± 0.2	*< 0.12*	0.4 ± 0.3	40 ± 30	*< 5.2*	100 ± 100	*< 4.5*	3 ± 1	70 ± 30	2 ± 1	*< 2.3*	*< 0.8*	4 ± 2	*< 4.6*	0.9 ± 0.3	*< 0.055*	0.5 ± 0.2	80 ± 30
Tobacco	16to	Liban	70 ± 10	7 ± 2	*< 37*	30 ± 10	*< 0.057*	*< 0.089*	*< 0.12*	0.4 ± 0.2	40 ± 30	7 ± 7	800 ± 500	*< 4.5*	1.4 ± 0.5	6 ± 2	*< 0.45*	*< 2.3*	*< 0.8*	6 ± 3	*< 4.6*	0.5 ± 0.2	0.06 ± 0.03	0.6 ± 0.2	140 ± 40
Tobacco	17to	Lucky	*< 26*	6 ± 2	100 ± 60	15 ± 10	*< 0.057*	0.2 ± 0.1	*< 0.12*	0.8 ± 0.5	30 ± 20	*< 5.2*	120 ± 90	*< 4.5*	2.1 ± 0.9	*< 1.6*	3 ± 2	*< 2.3*	*< 0.8*	4 ± 2	*< 4.6*	*< 0.24*	*< 0.055*	*< 0.21*	70 ± 20
Tobacco	18to	Nft	*< 26*	11 ± 5	90 ± 50	45 ± 10	*< 0.057*	0.2 ± 0.2	*< 0.12*	0.3 ± 0.2	30 ± 20	*< 5.2*	100 ± 100	*< 4.5*	1.3 ± 0.5	*< 1.6*	2 ± 1	*< 2.3*	*< 0.8*	4 ± 2	*< 4.6*	0.4 ± 0.1	0.15 ± 0.6	0.5 ± 0.1	*< 62*
Tobacco	19to	Old Virginia	*< 26*	2 ± 1	50 ± 30	*< 15*	*< 0.057*	0.20 ± 0.09	*< 0.12*	0.1 ± 0.1	40 ± 30	*< 5.2*	600 ± 600	*< 4.5*	1.7 ± 0.7	5 ± 2	*< 0.45*	*< 2.3*	*< 0.8*	4 ± 2	*< 4.6*	*< 0.24*	*< 0.055*	*< 0.21*	*< 62*
Tobacco	20to	Pulp	*< 26*	5 ± 2	90 ± 50	*< 15*	*< 0.057*	0.1 ± 0.1	*< 0.12*	0.6 ± 0.3	30 ± 20	*< 5.2*	1500 ± 1000	*< 4.5*	2.0 ± 0.8	10 ± 4	*< 0.45*	*< 2.3*	*< 0.8*	3 ± 2	*< 4.6*	*< 0.24*	0.06 ± 0.03	0.6 ± 0.2	*< 62*
Tobacco	21to	Sibannac	*< 26*	6 ± 2	*< 37*	15 ± 10	*< 0.057*	0.15 ± 0.08	*< 0.12*	0.8 ± 0.5	40 ± 30	9 ± 9	700 ± 400	*< 4.5*	9 ± 4	40 ± 10	0.5 ± 0.4	7 ± 4	*< 0.8*	3 ± 1	*< 4.6*	0.3 ± 0.1	0.10 ± 0.05	*< 0.21*	220 ± 70
Tobacco	22to	T.Blended	*< 26*	6 ± 3	*< 37*	30 ± 10	*< 0.057*	0.1 ± 0.2	*< 0.12*	0.7 ± 0.4	40 ± 30	14 ± 8	120 ± 110	*< 4.5*	2.0 ± 0.8	3 ± 1	2 ± 1	6 ± 4	*< 0.8*	2 ± 1	*< 4.6*	0.5 ± 0.2	*< 0.055*	*< 0.21*	70 ± 20
Tobacco	23to	T.Ry4	*< 26*	5 ± 2	*< 37*	22 ± 7	*< 0.057*	0.1 ± 0.1	*< 0.12*	0.2 ± 0.2	30 ± 20	6 ± 4	400 ± 300	*< 4.5*	1.0 ± 0.4	4 ± 2	1 ± 1	3 ± 2	*< 0.8*	3 ± 2	*< 4.6*	0.5 ± 0.2	*< 0.055*	*< 0.21*	70 ± 20
Tobacco	24to	Vsca	*< 26*	1.5 ± 0.6	*< 37*	*< 15*	*< 0.057*	*< 0.089*	*< 0.12*	0.1 ± 0.1	30 ± 20	*< 5.2*	400 ± 400	14 ± 9	1.2 ± 0.5	3 ± 1	0.5 ± 0.4	*< 2.3*	1.3 ± 0.5	1.3 ± 0.7	*< 4.6*	0.28 ± 0.08	0.06 ± 0.03	*< 0.21*	70 ± 20
Tobacco	25to	Wt	*< 26*	5 ± 2	*< 37*	18 ± 5	0.06 ± 0.04	*< 0.089*	*< 0.12*	0.4 ± 0.2	30 ± 20	*< 5.2*	*< 53*	*< 4.5*	1.9 ± 0.8	40 ± 20	0.5 ± 0.4	*< 2.3*	1.0 ± 0.4	5 ± 2	*< 4.6*	0.3 ± 0.1	0.10 ± 0.05	*< 0.21*	70 ± 20
Tonic	26tn	Caramel	*< 26*	8 ± 3	*< 37*	26 ± 8	*< 0.057*	*< 0.089*	*< 0.12*	0.3 ± 0.2	30 ± 20	*< 5.2*	1000 ± 700	5 ± 4	1.1 ± 0.4	20 ± 8	0.6 ± 0.5	*< 2.3*	1.0 ± 0.5	6 ± 3	*< 4.6*	0.4 ± 0.1	0.10 ± 0.05	0.7 ± 0.2	70 ± 20
Tonic	27tn	Coffee Break	*< 26*	6 ± 2	80 ± 50	17 ± 5	*< 0.057*	*< 0.089*	*< 0.12*	0.3 ± 0.2	40 ± 30	*< 5.2*	60 ± 60	*< 4.5*	1.4 ± 0.5	1.6 ± 0.6	*< 0.45*	4 ± 2	*< 0.8*	10 ± 5	*< 4.6*	1.5 ± 0.6	0.10 ± 0.05	*< 0.21*	110 ± 30
Tonic	28tn	Dark Choc	*< 26*	10 ± 4	60 ± 40	18 ± 5	*< 0.057*	*< 0.089*	*< 0.12*	0.3 ± 0.2	30 ± 20	*< 5.2*	*< 53*	*< 4.5*	0.7 ± 0.3	1.6 ± 0.6	*< 0.45*	*< 2.3*	*< 0.8*	5 ± 3	*< 4.6*	*< 0.24*	*< 0.055*	*< 0.21*	70 ± 20
Tonic	29tn	Energy	*< 26*	6 ± 2	*< 37*	*< 15*	*< 0.057*	*< 0.089*	*< 0.12*	*< 0.089*	30 ± 20	*< 5.2*	90 ± 80	*< 4.5*	1.7 ± 0.7	4 ± 2	*< 0.45*	*< 2.3*	*< 0.8*	2 ± 1	*< 4.6*	*< 0.24*	*< 0.055*	*< 0.21*	120 ± 40
Tonic	30tn	Ice	*< 26*	10 ± 4	110 ± 70	*< 15*	*< 0.057*	*< 0.089*	*< 0.12*	0.6 ± 0.4	40 ± 20	*< 5.2*	*< 53*	*< 4.5*	2.2 ± 0.9	3 ± 1	3 ± 2	6 ± 3	*< 0.8*	3 ± 1	*< 4.6*	*< 0.24*	0.15 ± 0.07	*< 0.21*	130 ± 40
Tonic	31tn	Nice	*< 26*	8 ± 3	60 ± 40	*< 15*	*< 0.057*	*< 0.089*	*< 0.12*	*< 0.089*	20 ± 10	*< 5.2*	*< 53*	*< 4.5*	0.7 ± 0.3	*< 1.6*	*< 0.45*	*< 2.3*	*< 0.8*	*< 1.1*	*< 4.6*	0.3 ± 0.1	0.15 ± 0.07	*< 0.21*	170 ± 50
Tonic	32tn	Noir	*< 26*	3 ± 1	60 ± 40	*< 15*	*< 0.057*	*< 0.089*	0.12 ± 0.09	0.1 ± 0.1	40 ± 30	*< 5.2*	90 ± 80	*< 4.5*	1.8 ± 0.7	*< 1.6*	*< 0.45*	*< 2.3*	*< 0.8*	3 ± 2	*< 4.6*	0.3 ± 0.1	0.06 ± 0.03	*< 0.21*	90 ± 30
Tonic	33tn	Only Rum	*< 26*	3 ± 1	80 ± 50	*< 15*	*< 0.057*	*< 0.089*	*< 0.12*	0.3 ± 0.2	30 ± 20	*< 5.2*	*< 53*	*< 4.5*	1.3 ± 0.5	1.7 ± 0.5	1 ± 1	4 ± 3	*< 0.8*	*< 1.1*	*< 4.6*	*< 0.24*	*< 0.055*	*< 0.21*	130 ± 40
Tonic	34tn	Tiramisu	*< 26*	8 ± 3	100 ± 60	30 ± 9	*< 0.057*	*< 0.089*	*< 0.12*	0.6 ± 0.4	40 ± 30	6 ± 3	*< 53*	*< 4.5*	2.1 ± 0.8	10 ± 4	2 ± 2	*< 2.3*	*< 0.8*	1.4 ± 0.9	*< 4.6*	0.3 ± 0.1	0.06 ± 0.03	0.3 ± 0.1	*< 62*
Tonic	35tn	Vaniglia Dry	*< 26*	0.8 ± 0.4	*< 37*	*< 15*	*< 0.057*	*< 0.089*	*< 0.12*	*< 0.089*	30 ± 20	*< 5.2*	*< 53*	*< 4.5*	1.6 ± 0.6	1.6 ± 0.6	*< 0.45*	*< 2.3*	*< 0.8*	*< 1.1*	*< 4.6*	*< 0.24*	0.06 ± 0.03	*< 0.21*	90 ± 30
Tonic	36tn	X Tè	*< 26*	1.0 ± 0.4	*< 37*	*< 15*	*< 0.057*	*< 0.089*	*< 0.12*	*< 0.089*	30 ± 20	*< 5.2*	*< 53*	*< 4.5*	2.2 ± 0.9	5 ± 2	*< 0.45*	*< 2.3*	*< 0.8*	*< 1.1*	*< 4.6*	*< 0.24*	0.06 ± 0.03	*< 0.21*	110 ± 30
Tonic	37tn	Yin Yang	*< 26*	5 ± 2	140 ± 80	16 ± 5	*< 0.057*	*< 0.089*	*< 0.12*	0.5 ± 0.3	20 ± 10	7 ± 7	*< 53*	*< 4.5*	1.1 ± 0.4	7 ± 3	*< 0.45*	6 ± 3	*< 0.8*	6 ± 3	*< 4.6*	*< 0.24*	0.06 ± 0.03	*< 0.21*	*< 62*

Data were rounded according to the standard deviation. Concentration in $\mu g\ kg^{-1}$; each sample was analysed twice. Data reported in *italic* are below the LoD, data reported in underlined are below the LoQ.
